# The Dark Side of the Moon: The Right Ventricle

**DOI:** 10.3390/jcdd4040018

**Published:** 2017-10-20

**Authors:** Massimiliano Foschi, Michele Di Mauro, Fabrizio Tancredi, Carlo Capparuccia, Renata Petroni, Luigi Leonzio, Silvio Romano, Sabina Gallina, Maria Penco, Mario Cibelli, Antonio Calafiore

**Affiliations:** 1 Department of Heart Disease, SS Annunziata Hospital, 66100 Chieti, Italy; foschimassimiliano@libero.it (M.F.); fabriziotancredi@yahoo.it (F.T.); cardiostore@gmail.com (C.C.); luigi.leonzio@asl2abruzzo.it (L.L.); 2 Chair of Cardiology, University of L’Aquila, 67100 L’Aquila, Italy; renata.petroni@gmail.com (R.P.); silvio.romano@cc.univaq.it (S.R.); maria.penco@cc.univaq.it (M.P.); 3 Cardiology, University “Gabriele D’Annunzio”of Chieti-Pescara, 66100 Chieti, Italy; sgallina@unich.it; 4 Department of Cardiothoracic Anaesthesia, University Hospitals Birmingham, Birmingham B15 2TH, UK; mario.cibelli@uhb.nhs.uk; 5 Fondazione Giovanni Paolo II, 86100 Campabasso, Italy; am.calafiore@gmail.com

**Keywords:** right ventricle, right ventricular function, right ventricular failure

## Abstract

The aim of this review article is to summarize current knowledge of the pathophysiology underlying right ventricular failure (RVF), focusing, in particular, on right ventricular assessment and prognosis. The right ventricle (RV) can tolerate volume overload well, but is not able to sustain pressure overload. Right ventricular hypertrophy (RVH), as a response to increased afterload, can be adaptive or maladaptive. The easiest and most common way to assess the RV is by two-dimensional (2D) trans-thoracic echocardiography measuring surrogate indexes, such as tricuspid annular plane systolic excursion (TAPSE), fractional area change (FAC), and tissue Doppler velocity of the lateral aspect of the tricuspid valvular plane. However, both volumes and function are better estimated by 3D echocardiography and cardiac magnetic resonance (CMR). The prognostic role of the RV in heart failure (HF), pulmonary hypertension (PH), acute myocardial infarction (AMI), and cardiac surgery has been overlooked for many years. However, several recent studies have placed much greater importance on the RV in prognostic assessments. In conclusion, RV dimensions and function should be routinely assessed in cardiovascular disease, as RVF has a significant impact on disease prognosis. In the presence of RVF, different therapeutic approaches, either pharmacological or surgical, may be beneficial.

## 1. The Right Ventricle

The RV has a unique crescent shape which influences its physiological properties. The normal range of right ventricular end-diastolic volume (RVESV) is 49–101 mL/m^2^ (55–105 mL/m^2^ in men and 48–87 mL/m^2^ in women), whereas the normal range of left ventricular end-diastolic volume (LVESV) is 44–89 mL/m^2^ (men, 47–92 mL/m^2^; women, 41–81 mL/m^2^), assessed by MRI. In the normal adult, the mass of the RV is also only about one-third that of the left ventricle (LV): 26 ± 5 (17–34) g/m^2^ for the RV versus 87 ± 12 (64–109) g/m^2^ for the LV [1,2,3].

The RV encompasses two myocardial bands: the ventriculo-infundibular band; a muscular fold between the pulmonary valve and the tricuspid valve (TV) that extends to the subpulmonary infundibulum of the RV outlet, and the septomarginal band, extending from the septum to the anterior wall and supporting a papillary muscle. RV contraction is peristaltic, from the sinus to the conus, with a major radius of curvature of roughly 4 cm and a small radius of curvature of 0.8 cm [4]. The RV is composed of superficial (circumferential) and deep muscle layers (longitudinal) [5,6]. This arrangement contributes to the more complex movement of the, which includes torsion, translation, rotation, and thickening [5,6]. The continuity between the muscle fibers of the RV and LV contributes, along with the interventricular septum and pericardium, to ventricular interdependence [6].

The RV propels blood into the pulmonary circulation; a huge territory that has low hydraulic impedance. As a consequence, the thickness of the free wall in the RV is lower than in the LV. In the LV, there is a third layer of fibres (circumferential constrictor fibres) which account for the reduction of ventricular diameter. The RV, lacking this layer, has to rely more on longitudinal shortening than the LV. RV contractions mainly depend on its loading conditions.

The pressure-volume relationship of the RV was defined by Redington et al. [7] as a triangular or trapezoidal shape. Thus, unlike the square wave pump of the LV, the RV is an energy-efficient pump (with a myocardial energy cost of approximately one-fifth of that of the LV), which is almost entirely dependent on the low pulmonary hydraulic impedance. In fact, when the LV is beneath the pulmonary artery, as in corrected transposition of great arteries, its pressure-volume characteristics are identical to those of the normal RV [8,9].

Kovalova et al. [3] found that the right ventricular ejection fraction (RVEF) was constantly lower than the left ventricular ejection fraction (LVEF), but the difference was more pronounced in men (50.0% ± 9.7 vs. 60.7% ± 8.4) than in women (58.0% ± 13.6 vs. 61.7% ± 9.4).

The ventricles share not only the visceral cavity (pericardium) but also myofibres, particularly in their superficial layers, and the interventricular septum, which contributes to the ejection of both cavities. Damiano et al. [10] suggested that roughly 30% of the contractile energy of the RV was generated by the LV. The LV positively influences RV performance, contributing significantly to its pressure generation, both as a whole and through septal contraction. Conversely, the RV has little or no effect on LV pressure generation. When there is RV enlargement and reduction of RV free wall contractility, there is a progressive reduction in both RV and LV mechanical work; thus, LV pressure development and stroke work dcrease. Danton et al. showed that acute RV ischaemia due to coronary artery ligation, induced LV dysfunction [11].

## 2. Adaptive and Maladaptive Hypertrophy

RVF can result from global or regional ischaemia due to coronary disease, but most commonly it is the result of increased afterload. In fact, even mild increases in afterload can lead to profound decreases in RV stroke volume [12]. In some cases, chronic volume overload can lead to RVF as well. Compared with the LV, the shape and the reduced thickness of the RV make it more prone to volume overload when preload increases. When afterload increases for any reason, the wall stress increases at the same time, therefore, the RV has to become thicker (by accumulating muscle mass) thereby assuming a more rounded shape.

**Figure 1:**
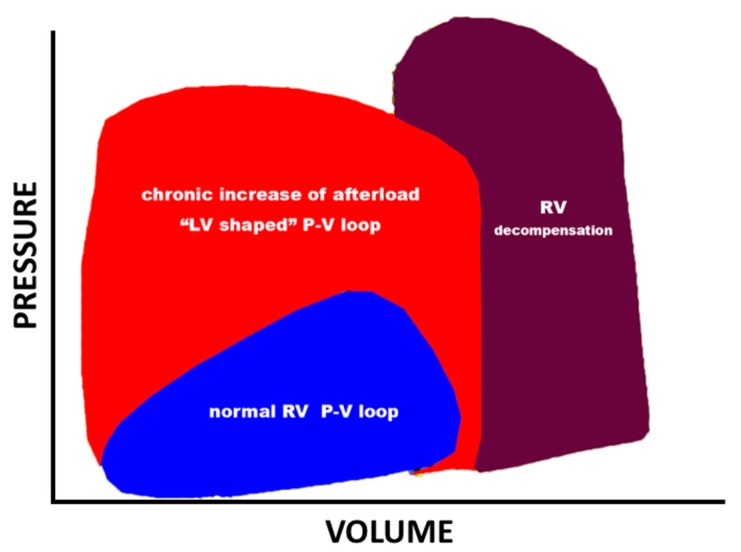
Right ventricular (RV) pressure-volume (P-V) loops obtained by a catheterism. The blue loop depicts a normal RV P-V loop. The red loop represents a compensated, chronically hypertensive RV (left ventricle, LV-shaped loop). The violet loop represents a decompensated RV.

The RV pressure-volume pattern (being related to low flow impedance) is a dynamic phenomenon in which a slowly progressive rise in pulmonary arterial impedance causes a progressive change towards a LV-type pressure–volume loop [13] (Figure 1).

Right ventricular hypertrophy (RVH), as a response to increased afterload, can be either adaptive (concentric hypertrophy, minimal dilatation or fibrosis, normal exercise capacity, ejection fraction, and output) or maladaptive (dilatation, fibrosis, reduced exercise capacity, ejection fraction, and output). In some cases, increased RV mass is sufficient to compensate for increased afterload while maintaining an unchanged RV cardiac output. When insufficient, increased RV mass leads to a maladaptive response and rapid decompensation [13,14]. The molecular features of adaptive and maladaptive RVH have recently been reviewed [15]. There are many factors determining the type of hypertrophy, for example: cellular and molecular changes, ischaemia, fibrosis, and autonomic dysregulation. Animal models of RVH have demonstrated that the heterogeneity of RVH cannot be fully explained simply by differences in RV mass or pressure overload [16].

Moreover, coronary perfusion may fail to meet the increased demands for oxygen in RVH, causing ischaemia, and leading to RVF [17] Right coronary flow is reduced when RV pressure overload causes excessive intramural increase in right ventricular pressure. This may also occur in diastole resulting in ischaemia and RV contractile function reduction [18,19]. Moreover, during the development of cardiac hypertrophy, a mismatch between the number of capillaries and the size of cardiomyocytes can lead to myocardial hypoxia, contractile dysfunction, and apoptosis [20,21].

## 3. Right Ventricular versus Left Ventricular Failure

In chronically increased afterload, the RV pressure-volume relationship shifts from a trapezoidal shape (reflecting its high efficiency/low impedance) to a square or rectangular shape with well-developed isovolumic contraction and relaxation periods rendering the normal LV pressure-volume loop indistinguishable from that of the RV (Figure 1).

Nowadays, it is well-known that the main differences between the two ventricles are not limited to global structure and loading conditions, as early during cardiac development LV cardiomyocyte differentiation begins to diverge from that of the RV [22].

In fact, differences at the cellular and molecular levels form the basis of differential right versus left ventricular responses to pressure overload stress. Both ventricles exhibit similar alterations in the genes regulating the extracellular matrix and cytoskeletal remodeling, but with significant differences in those genes regulating energy production, mitochondrial function, reactive oxygen species (ROS) production, antioxidant protection, and ischaemia [23,24,25]. From an energetic perspective, both the right and left ventricles show a metabolic shift to glycolysis as a response to afterload stress, but this switch occurs earlier in the RV than in the LV, causing a drop in ATP production to occur sooner in the RV. Under afterload stress, both ventricles show an increase in ROS production, but the RV antioxidant defenses fail early, whereas in the LV they remain intact until a more advanced stage of failure [26,27].

In patients with PAH, leftward displacement of the interventricular septum hampers LV filling [28].

## 4. Causes of RVF

There are several known causes of RVF, but this condition is generally attributed to PAH, TV disease, left-side heart failure with secondary pulmonary hypertension, chronic pulmonary disease, left ventricular assist device (LVAD) implantation, or congenital heart diseases.

### 4.1. RVF and PAH

PAH is a type of pre-capillary pulmonary hypertension, characterized by normal or low wedge pressure [29], which causes a pressure overload and a consequential adaptive hypertrophy of the RV. At a compensated stage, the RV shows normal or even reduced right ventricular wall stress and such patients can be totally asymptomatic. However, the RV is not able to compensate for too long, and soon the adaptive hypertrophy becomes maladaptive; accompanied by fibrosis, diastolic and systolic dysfunction, RV dilatation, higher wall stress, and higher oxygen consumption. The rising end-diastolic pressure, along with the higher RV mass, leads to ischaemia and worsening RVF. In addition, dilatation of the RV causes functional tricuspid regurgitation that further compromises forward output and causes RV volume overload as well. Progressive right ventricular dilation, in the setting of pericardial constraint and diastolic ventricular interdependence, compromises left ventricular filling via several different mechanisms [30].

The decompensated phase of right ventricular systolic failure produces symptoms with minimal activity or at rest. Elevated right atrial pressure and systemic venous hypertension cause hepatic congestion, leading to an enlarged, pulsatile liver and ascites. Renal venous congestion, combined with decreased renal arterial perfusion causes diuretic resistance, reduced urine output, and prerenal azotemia [31]. A low cardiac output state is also evident, resulting in fatigue and syncope or pre-syncope.

### 4.2. RVF and Tricuspid Valve Regurgitation

Tricuspid valve regurgitation (TR) is most commonly secondary to left ventricular valve disease, and is mainly consequent to mitral valve disease. More rarely, TR occurs as a result of valve endocarditis, rheumatic valve disease, carcinoid or congenital heart disease. The RV and the tricuspid valve have a bidirectional relationship, as TR can cause RV failure and RV failure can cause TR. Functional tricuspid regurgitation (FTR), (defined as secondary TR with normal leaflets), is the result of both annular dilatation and leaflet tethering due to RV enlargement, factors which can themselves also influence leaflet coaptation via different mechanisms.

The annular enlargement develops mainlyat the level of the free wall and is accompanied by changes in planar shape. Ton-nu et al. [32] found that the three-dimensional aspect of TV shape was flattened in patients with significant TR and that there was an inverse and continuous relationship between the annular area and the degree of planarity.

However, annular dilatation alone is insufficient to cause TR, even if it can impair proper coaptation of the leaflets. In an interesting study, Sadeghi et al. [33] reported 27 patients with pulmonary thromboembolism who had severe PH and severe TR. They underwent surgery without TV annuloplasty. In 19 patients (70%), the pulmonary pressure dropped by a mean of 49 mmHg, and TR was reduced to a mild state. In the remaining 30% of patients, TR remained severe; with a smaller (32 mmHg) reduction in pulmonary pressure compared with the first group. Interestingly, the annular size remained nearly unchanged in both groups; with a 4 mm reduction between pre- and post-operative echocardiograms and similar measurements (41 mm in the first group and 42 mm in the second group) at the post-operative assessment.

RV dilatation causes an inward displacement toward the centrum of the three papillary muscles (PMs), caused by the displacement of the septal wall into the RV as occurs with increased LV volume. Changes in LV geometry are transferred to the RV though the interventricular septum [34], resulting in a configuration that may lead to TV leaflet prolapse [35]. When the septum is bowing towards the RV, the mobility of the septal leaflet is increased, thus changing the location of the coaptation line resulting in central defectivecoaptation, due to functional shortening of the septal leaflet. In this context FTR fuels more FTR and this, in turn, can lead to RV volume overload and liver congestion.

### 4.3. RVF and Left Ventricular Failure

The interdependence of RV and LV is the root cause of biventricular failure at end-stage heart failure. In the case of increased RV afterload, RV systole is prolonged, extending into LV diastole, and is worsened by increasing heart rate. Hence, RV filling time is severely shortened [28], compromising biventricular filling rate. Thus, the overall systolic time lengthens but RV ejection time shortens and filling time decreases significantly. The shortened ejection time and reduced stroke volume, secondary to increased afterload, further contribute to decreased LV filling and cardiac output [36].

The interaction between the two ventricles is particularly evident when LV is mechanically unloaded. Mau et al. [37] demonstrated that ≥75% of LV unloading induces LV volume reduction and RV volume expansion that contributes to load-independent RV dysfunction and leads to the deterioration of haemodynamic function. This results in RV failure. This is due to impaired septal compliance resulting from septal fibrosis that limits movement into the LV cavity. On the other hand, if the septum maintains its function, LV unloading not only causes the modification of RV geometry, but also reduces RV output by reducing septal thickening, contributing to RV failure during mechanical LV support [38].

Most of the effects of LV contraction on the RV are mediated by the interventricular septum. During systole, the septum twists and shortens, causing a reduction in ventricular volume and forceful ejection of blood out of both ventricular cavities. In the RV, in the absence of septal twisting due to septal damage, ventricular ejection is produced by circumferential constriction caused by the contraction of the basal wall that contains predominantly transverse fibres. Such constriction may not allow for the delivery of enough contractile force to ensure adequate cardiac output when pulmonary vascular resistance is increased. Septal dysfunction may form the basis of RV dysfunction that develops perioperatively, together with new septal akinesia or hypokinesia.

When the RV is dilated, the interventricular septum shifts toward the left, altering LV geometry and increasing pericardial constriction. As a consequence, LV preload is decreased and LV end-diastolic pressure is increased, causing low cardiac output states [39,40]. When the initial event is LV failure, then the septal contribution to RV function is impaired and RV ejection becomes predominantly dependent on the transverse fibres of the basal loop. Elevation of pulmonary artery pressure occurs because of increased LV end-diastolic pressure and results in RV failure. Septal stretching and the bowing toward the contralateral cavity that results, then becomes the underlying cause of impaired biventricular performance. The timing of septal contraction is also important. Impaired excitation contraction coupling, arising from electrical signal malfunctions (i.e., wide QRS complex and bundle branch block) similarly impairs the action of a viable and well-perfused septum. Thus, it is not only important for the septum to be contractile, but this contraction must be sequential and occur at the right time during the cardiac cycle, in order to maximize the effective septal contribution to blood ejection.

The interdependence between right and left ventricles is the basis for the use of an intra-aortic balloon pump (IABP) in cases of RVF. Left diastolic dysfunction related to septal bowing to the left can improve after IABP positioning, and also causes an indirect positive effect on RV performance [41].

### 4.4. RVF and Chronic Pulmonary Disease

According to World Health Organization (WHO), the majority of cases of PH and RVF are those associated with chronic lung diseases; such as chronic obstructive pulmonary disease (COPD), interstitial lung disease (ILD), and sleep disordered breathing (SDB) [42].

The pathophysiological changes underlying RVF in chronic pulmonary disease are increased pulmonary vascular resistance (PVR), resulting in the pulmonary arterial pressure rising roughly 3 mmHg/year and RV pressure overload. Chronic hypoxaemia and the disruption of pulmonary vascular beds through parenchymal loss and fibrosis, are the key mechanisms through which chronic lung disease increases PVR. As for PAH, concentric hypertrophy of the RV is an adaptive mechanism to preserve systolic function [43], but this can become maladaptive with time, leading to right ventricular eccentric hypertrophy and systolic failure. RV diastolic function may also be impaired in chronic lung disease patients with PH. In these patients, a direct association has been demonstrated between PH, reduced early to late ventricular filling velocity ratio (E/A ratio), and prolonged myocardial relaxation time. The prevalence of PH ranges from 30% to 70% in COPD, from 8% to 40% in ILD, and from 20% to 40% in SBD [44].

### 4.5. RVF and after LVAD Implantation

RVF represents a frequent complication following LVAD implantation; with the incidence of RVF ranging from 20% to 50% of LVAD cases. Although, in theory, RVF may benefit from a reduction of left atrium pressure, leftward intraventricular septum (IVS) shift and increased venous return, may lead to RV dysfunction [45]. The deterioration in RV function is indicated by an increase in RV size and the severity of TR, as compared with baseline measurements. In this particular subset of patients, RVF is defined as the inability of the pulmonary circulation to fill the LVAD. The diagnostic criteria for RVF include the signs and symptoms of persistent right ventricular dysfunction: a central venous pressure (CVP) of >18 mmHg with a cardiac index (CI ≤ 2.0 L/min/m^2^) in the absence of an elevated left atrial/pulmonary capillary wedge pressure (PCWP) >18 mmHg; tamponade; ventricular arrhythmias or pneumothorax requiring right ventricular assist device (RVAD) implantation or requiring inhaled nitric oxide or inotropic therapy for a duration of more than one week at any time after LVAD implantation; ascites or evidence of moderate-to-severe peripheral oedema; evidence of elevated CVP by echocardiogram (dilated inferior vena cava without collapse); and signs of increased jugular venous pressure during the physical exam. The optimal haemodynamic parameters of preoperative RV function, indicating a low probability of developing RVF, are CVP ≤ 8 mmHg; PCWP ≤ 18 mmHg; CVP/PCWP ≤ 0.66; PVR < 2 wood units; and right ventricular stroke work index (RVSWI) ≥ 400 mmHg mL/m^2^ [46].

### 4.6. RVF and Congenital Heart Disease

RVF remains an important clinical problem in several congenital heart diseases (CHDs). Pressure-overload RV failure is caused by RV outflow tract obstruction after total correction of tetralogy of Fallot, pulmonary stenosis, atrial switch operation for transposition of the great arteries, congenitally corrected transposition of the great arteries, and systemic RV failure after the Fontan operation. Volume-overload RV failure may be caused by an atrial septal defect, pulmonary regurgitation, or tricuspid regurgitation [47].

Isolated pulmonary stenosis is the most common right ventricular outflow tract (RVOT) obstructive CHD. Although the obstruction may also occur at the subvalvar or supravalvar levels, 80% to 90% of cases have valve-level obstruction [48]. Regardless of the level of obstruction, the RV exerts a hypertrophic response according to the degree of obstruction [49].

In moderate-to-severe pulmonary valve stenosis, patients remain asymptomatic until adulthood. The right ventricle is able to adapt to pulmonary valve stenosis. However, long-lasting untreated severe obstruction may lead to RV failure and tricuspid regurgitation. Congenitally corrected transposition of great vessels (TGA) is associated with moderate-to-severe systemic atrioventricular valve (tricuspid valve) regurgitation and RVF [47].

The RV adapts better to volume overload than to pressure overload [50]; however, chronic volume overload is associated with RVF, as in the case of large atrial septal defect (ASD), which evolves to Eisenmenger's syndrome with pulmonary vasculopathy development. Severe pulmonary regurgitation is the most common cause of progressive RV dilatation and dysfunction in patients with repaired tetralogy of Fallot. The Ebstein anomaly is characterized by an apical displacement of the septal and posterior tricuspid leaflets exceeding 8 mm/m^2^, leading to an atrialized RV and moderate-to-severe tricuspid regurgitation. RVF in the Ebstein anomaly results from volume overload of the RV and from a hypoplastic RV chamber unable to manage the systemic venous blood [50].

## 5. The Prognostic Role of RVF

The prognostic role of RVF in LV heart failure (HF), PH, acute myocardial infarction (AMI), and cardiac surgery has been overlooked for many years. However, more recently, several studies have attributed a more prominent role to the RV in prognostic assessments [51,52,53,54,55,56,57,58,59,60,61,62,63,64,65,66,67,68,69,70,71,72,73,74]. Recent studies have shown that RV function is a significant, independent predictor for outcomes in patients with left ventricular dysfunction complicating AMI. In particular, the involvement of the RV due to inferior AMI is a strong predictor of major complications and in-hospital mortality. Right ventricular dysfunction is also common in patients with anterior infarction, especially when moderate-to-severe left ventricular dysfunction is present [51,52,53,54,55,56,57,58,59].

In a recent study, the roles of PH and right ventricular dysfunction were evaluated in patients with AMI and LV dysfunction. The impact of RV dysfunction without PH on mortality was restricted to the first 30 days after the infarct, without any effect on long-term mortality or the risk of re-admission for HF. Beyond the 30-day timepoint, PH was the dominant risk factor for long-term mortality. In addition, PH (a surrogate of increased left atrial pressures), was a strong independent predictor of re-admission for HF, whereas RV dysfunction without PH was not [69].

Right ventricular dysfunction may develop in association with left ventricular dysfunction via a number of different mechanisms listed here. Firstly, left ventricular failure increases afterload by increasing pulmonary venous and ultimately pulmonary arterial pressure; the same cardiomyopathy may simultaneously affect both ventricles; myocardial ischaemia may involve both ventricles; left ventricular dysfunction may lead to decreased right ventricular coronary perfusion, which may be an important factor influencing onright ventricular function; and lastly, ventricular interdependence due to septal dysfunction may occur so that left ventricular dilatation in a limited pericardial compartment may restrict right ventricular diastolic function. In patients with advanced congestive heart failure due to cardiomyopathy or ischaemia, right ventricle shortening is the only significant independent factor found by multivariate analysis to be associated with survival (in contrast to other parameters tested, including: left ventricular ejection fraction, cardiac index, and pulmonary resistance). Patients with right ventricular shortening <1.25 cm have a significantly worse actuarial survival over two years (16% versus 68% for >1.25 cm) [70]. Preserved right ventricular function in patients with severe congestive heart failure and PH confers a survival advantage comparable to that experienced by patients without PH. However, in this study, reduced right ventricular ejection fraction alone, in the absence of PH, did not exhibit an additional survival risk [60]. PH is equally prevalent in HF with or without reduced ejection fraction (EF) [71,72]. Conversely the prevalence of RVD in HF with preserved and reduced EF (HFpEF and HFrEF, respectively) is not well established. Puwanant and Damy described a 1.5- to 2-fold higher prevalence of RVD in HFrEF compared with HFpEF [72,73].

RV dysfunction has been found to be an independent risk factor for both overall and cardiovascular mortality in HFpEF with eight years of follow-up [74].

In cardiac surgery, although there is sufficient evidence in favour of a routine pre-operative assessment of the RV [63,64,65,66,67,68], the main surgical risk score systems don’t consider either RV dysfunction or dilatation.

In contrast, PH is considered to be a strong predictor for mortality after cardiac surgery. However, as has been already demonstrated in patients with HF, PH does not always mirror RV dysfunction, which has an independent and additive prognostic value [58]. In addition, RV dysfunction seems to be a better predictor of post-operative circulatory failure, rather than PH, in patients undergoing mitral valve surgery [63,64].

Following cardiac surgery with cardiopulmonary bypass and pericardial incision, patients show a marked change in the RV contractile pattern, with a relative loss of longitudinal shortening and a gain in transverse shortening. This can occur as a result of a poor myocardial protection or intra-operative myocardial infarction due to early graft failure, a prolonged donor ischaemic time, and a donor RV not adapted to elevated pulmonary vascular resistance in the recipient, as a consequence of end-stage HF.

## 6. The Assessment of Right Ventricular Failure 

The most common way to assess the RV is through two-dimensional trans-thoracic echocardiography (2D TTE). Although, it should be noted that the assessment of RV morphology and function is very difficult with this method due to the position of the RV immediately below the sternum.

However, the systematic assessment of the right side of the heart is still not routinely performed. This is due to the greater consideration given to left side of the heart during patient evaluation, a lack of familiarity with ultrasound techniques that can be employed in imaging the right side of the heart, and the rarity of studies reporting normal reference values for RV dimensions and function. Consequently, a recent review was published by the American Society of Echocardiography, and endorsed by the European Association of Echocardiography and the Canadian Society of Echocardiography, with the aim of proposing guidelines and standards for the assessment of the right side of the heart in adults [75].

The right ventricular chamber size can be assessed at end-diastole from the apical four-chamber view (A4CV) with the focus on the RV. Three different widths can be assessed: basal, mid-cavity, and longitudinal diameter. Another parameter to assess is the RV free wall thickness, measured at end-diastole in subcostal view, at the level of the tip of the anterior tricuspid leaflet; usually an RV free wall thickness >5 mm is considered abnormal.

The estimation of right ventricular systolic function by means of ejection fraction (RVEF), derived from RV volumes in 2D echocardiography, shows several limitations mainly due to the particular shape of the RV [6,76,77]. In fact, 2D echocardiography underestimates RV volume with respect to either MRI or 3D echocardiography [78]. Hence, instead of RVEF, it is possible to assess some surrogate parameters such as RV fractional area (RV FAC), tricuspid annular plane systolic excursion (TAPSE), systolic excursion velocity of lateral tricuspid annulus by means of Tissue Doppler Imaging (TDI S’), myocardial acceleration during isovolumic contraction (RV IVA), regional RV strain, and strain rate. Fractional area change (defined as end-diastolic area—end-systolic area/end-diastolic area ×100) is calculated in A4CV and correlates with RVEF by MRI [78]. TAPSE is easily obtained from A4CV by placing an M-mode cursor at the level of the lateral part of the tricuspid annulus and measuring the amount of longitudinal motion of the annulus at the systolic peak [79]. TDI S’ represents the peak velocity of the tricuspid annulus during its systolic excursion, measured using pulsed tissue Doppler and colour-code tissue Doppler (the cursor should be placed at the level of the lateral part of the tricuspid annulus from an A4CV). Some cut-offs have been identified to define RV systolic dysfunction: FAC < 35%, TAPSE < 16 mm, and TDI S’ < 10 cm/s.

The evaluation of systolic pulmonary artery pressure with an estimation of peak tricuspid regurgitation (TRV) velocity and right atrial pressure derived from inferior vena cava size and collapse, should be routinely performed to complete the right ventricular heart echocardiographic assessment [75]. However, according to latest European Society of Cardiology ESC guidelines [29], transthoracic echocardiography should aim to assign a level of probability of PH. The same ESC guidelines suggest grading the probability of PH based on peak TRV at rest and on the presence of additional pre-specified echocardiographic variables suggestive of PH: low probability of PH when peak TRV is ≤2.8 m/s; intermediate probability with TRV ≤2.8 with other echo PH signs or TRV 2.9–3.4 m/s with other echo PH signs; high probability in the case of TRV 2.9–3.4 m/s with other echo PH signs or with TRV >3.4 m/s. Other echo PH signs include: RV dilation or flattening of the interventricular septum; dilated pulmonary artery; early diastolic pulmonary regurgitation velocity >2.2 m/s; Doppler acceleration time at the level of the RV outflow tract <10.5 m/s; inferior cava diameter >21 mm with decreased collapse; and right atrial area >18 cm^2^.

3D real-time transesophageal echocardiography is a very accurate way to assess RV volumes and RVEF, even if minimal data are available in either normal or dilated RV states. Moreover, both volumes and function are underestimated with 3D echocardiography with respect to cardiac magnetic resonance imaging (CMR). Although CMR is nowadays considered to be the most accurate imaging technique for the study of morphology, volumes, function, and tissue determination of the RV, it is still costly and not widely used.

## 7. The Treatment of RVF

RVF can be treated either medically or surgically. First, preload should be optimized and afterload must be reduced. Finally, myocardial contractility should be improved.

The most common conditions leading to RVF are characterized by high RV afterload. Hence, reducing excessive RV preload with diuretics or haemofiltration is necessary to reduce RV dilatation and free wall tension, minimizing RV ischaemia and optimizing contractility. Diuretics are recommended in patients with PAH or secondary PH [29]. Conversely, in patients with advanced RVF, preload is low due to reduced venous tone, vasoplegia, and positive pressure ventilation. In the case of normal afterload, preload should be increased to maintain forward flow. It is generally agreed that a right ventricular diastolic filling pressure of 8–12 mmHg is optimal in RVF.

Acidosis, hypoxia, and hypercapnia have to be corrected in order to reduce PVR as well as RV afterload. Pulmonary vasodilators include several classes of drugs that are approved for use in PAH. However, vasodilators have the potential to cause systemic hypotension. Only a small number of patients do well with calcium-channel blockers (CCBs). The most commonly used CCBs are nifedipine, diltiazem, and amlodipine, with nifedipine and diltiazem deemed most important [29,80]. The choice of CCB is based on the heart rate at rest, with a relative bradycardia favouring the use of nifedipine and amlodipine and a relative tachycardia favouring the use of diltiazem. CCBs are recommended only in patients with WHO functional class II and III (Level I C) [29]. Another class of drugs used in PAH are the endothelin receptor antagonists (ambrisentan, bosentan, macitentan) (Level I A or B). Phosphodiesterase 5 (PDE5) inhibitors (sidenafil, tadalafil, vardenafil, riociguat) block the degradation of cyclic guanosine monophosphate. They decrease pulmonary arterial pressure and increase cardiac output in both acute and chronic PH. Prostacyclin analogues (epoorstenol, iloprost, treprostil, beraprost) and prostacyclin receptor agonists (selexipag) act on prostacyclin, inducing potent vasodilation of all vascular beds [29].

In the acute decomposed phase, improving right ventricular contractility, can be achieved using low-dose dobutamine (5–10 µg/kg/min), which has been shown to restore cardiac output better than norepinephrine [81]. Dopamine also improves right ventricular contractility and, at doses below 16 µg/kg/min, it increases cardiac output without increasing PVR [81]. Dopamine would be preferable for hypotensive, non-tachycardic patients. Milrinone, a selective PDE3 inhibitor, slows intracellular cyclic adenosine monophosphate (cAMP) metabolism, improves inotropy, and facilitates pulmonary vasodilation. It is an attractive agent to use in PH resulting from biventricular failure [82] and would also be preferable to use in normotensive patients on chronic beta-blocker therapy. Inhaled milrinone has also been investigated for the management of RHF, as it avoids the side effect of systemic hypotension. In a randomized trial of high-risk cardiac surgery patients, inhaled milrinone was associated with increases in cardiac output and a reduction in pulmonary artery systolic pressure without causing systemic hypotension. Of note, however, these favourable haemodynamic effects did not translate into an improvement in clinically relevant endpoints [83,84].

Levosimendan, a calcium sensitizer, has been shown to improve RV function in left heart disease [85]. However, in a study comparing levosimendan with milrinone in patients undergoing cardiac surgery, levosimendan resulted in a greater increase in heart rate, a decrease in systemic vascular resistance, and a greater need for norepinephrine [86].

When RVF is caused by left ventricular failure, congenital heart disease, or tricuspid regurgitation, the identification and management of treatable causes of RVF is an important aspect of any management strategy. Thus, surgery can be an option to correct coronary artery disease and valve heart disease. In this review, we limited the discussion to the surgical correction of functional tricuspid regurgitation.

Surgical strategies to correct FTR are directed mainly to the annulus, using an incomplete ring or a band [87], or to the annulus and the leaflets (as in the Kay technique) [88]. Other techniques directed to the leaflets can be of interest in selected cases; including the edge-to-edge technique (associated with a rigid incomplete ring or a flexible band) [89] and leaflet augmentation [90]. Discussion of the latter technique notes that TV function is strictly correlated to the RV geometry, which depends on preload, on afterload, on LV geometry, and on ischaemic insults. It is clear also that the regression of right ventricular volumes to normal or near normal values is unpredictable, as different causes can have different outcomes [91]. As the mechanism of functional TR does not dwell only in the annulus, the surgical techniques usually applied (mainly to address annular reduction) have variable results. Navia et al. [92] reported long-term outcomes in 2277 patients who experienced different surgical techniques for annuloplasty, and where a few patients had surgery directed to the leaflets using the edge-to-edge technique. They found that 32% of patients were without TR at three months and 22% at five years, whereas the incidence of patients with 3+/4+ TR rose from 11% at three months to 17% at five years. 

In patients undergoing TV surgery, RV dilatation and severe tethering are frequently observed. In these circumstances, TV annuloplasty leads to increased tethering of the anterior and posterior leaflets, as the annulus is displaced towards the septum. As a consequence, in the presence of RV dilatation, surgical tethering will result in the worsening of post-operative FTR rather than better leaflet coaptation. The recurrence rate of moderate or greater FTR ranges from 2.5% to 5.5% at one-year follow-up [93].

In cases refractory to medical therapy or surgery, the timely deployment of mechanical circulatory support may offer a bridge to recovery or to definitive management of the underlying cause. Percutaneous support options include extracorporeal membrane oxygenation (ECMO), which offers right- and left-sided circulatory support. Currently available options for percutaneous right ventricular assist devices include the Impella^®^ RP System (Abiomed, Danvers, MA, USA), and TandemLife (CardiacAssist, Inc. Pittsburgh, PA, USA) using the PROTEK Duo™ cannula. Surgically cannulated pumps such as the Levitronix^®^ CentriMag^®^ left ventricular assist system (Levitronix LLC, Waltham, MA, USA) can also offer RV support, when employed in a right atrial and pulmonary artery configuration. Although none of the durable ventricular assist devices are currently approved for RV support, their use has been reported. In recent years, the need for RVAD during LVAD implantation has decreased significantly, as demonstrated in the INTERMACS report of 2001 the implantation rate has decreased from 24.7% in 2006 to 5% in 2011 and 2.9% in 2012, attributed to better patient selection for LVAD. Selected patients with refractory RHF may be candidates for transplantation or total artificial heart implants.

## 8. Conclusions

When the right ventricle fails, it does so mostly in the presence of pressure overload, as occurs in several cardiovascular diseases. RV failure has a significant impact on disease prognosis, therefore, RV dimensions and RV function should be routinely assessed during patient evaluation. In the presence of RV failure, different approaches, either pharmacological or surgical, may help improve patient outcomes.
